# Ultrasound assisted supramolecular liquid phase microextraction procedure for Sudan I at trace level in environmental samples

**DOI:** 10.3906/kim-2104-5

**Published:** 2021-10-19

**Authors:** Mustafa SOYLAK, Özgür ÖZALP, Furkan UZCAN

**Affiliations:** 1 Department of Chemistry, Faculty of Sciences, Erciyes University, Kayseri Turkey; 2 Technology Research & Application Center (TAUM), Erciyes University, Kayseri Turkey; 3 Turkish Academy of Sciences (TUBA), Ankara Turkey

**Keywords:** Liquid phase microextraction, environmental samples, separation-preconcentration, Sudan I, UV-Vis spectrophotometry

## Abstract

A method based on supramolecular liquid phase microextraction has been developed for the preconcentration and determination of trace levels of Sudan I. 1-decanol and tetrahydrofuran were used as supramolecular solvent components. Trace levels of Sudan I were extracted into the extraction solvent phase at pH = 4.0 Analytical parameters such as pH value, supramolecular solvent volume, ultrasonication, centrifugation, model solution volume, matrix effects have been optimized. The limit of detection and the limit of quantification values for Sudan I were calculated as 1.74 μg L^−1^ and 5.75 μg L^−1^, respectively. In order to determine the accuracy of the method, addition and recovery studies were carried out to environmental samples.

## 1. Introduction

It is known that about 10,000 different dyestuffs and pigments are used, and at least 10 % of them are thought to be biodegradable. Azo dyestuffs offer a wide range of colors and are used in many dyeing processes in the industry. The exposure to of these dyestuffs is generally through eating and skin absorption [1–3dye (RB5]. Among these dyes, Sudan dyestuffs are in the class of azo dyestuffs and are used to dye materials such as plastics, leathers and fabrics. Due to its interesting color and brightness, it is also illegally used to color spices such as chili and curry. Sudan I (1-phenylazo-2-naphthol) dye is a mutagen and is known to be carcinogenic to bladder and liver organs in mammals. Sudan I listed as category three carcinogens by International Agency for Research on Cancer (IARC) [4–10].

Sudan dyes has been determined so far by many methods such as ultra-high-performance liquid chromatography coupled with quadrupole time-of-flight tandem mass spectrometry (UHPLC-Q-TOF/MS) [11], high-performance liquid chromatography (HPLC) [12], capillary liquid chromatography (CLC) [13Sudan II, Sudan III and Sudan IV], ultrafast liquid chromatography (UFLC) [14]. However, these devices used in these methods are relatively complicated, expensive, relatively difficult to use, require consumables, and require long analysis processes. On the other hand, UV-Vis spectrophotometer is a device that is low-cost compared to these devices and can be found in almost every laboratory; it is very easy to use, does not require a lot of consumables, offers a fast analysis, does not require a trained operator [15,16]. However, due to problems such as interference effects and analytes being lower than the detection limits of the devices, a separation - preconcentration technique should be applied before analysis.

Microextraction methods use separation-preconcentration technique when compared to classical sample preparation techniques such as solid phase extraction and liquid phase extraction. Microextraction methods have many advantages such as use of low amounts of solvents that comply with the new generation green principles, ease of automation, ability to perform the process with simple instruments available in the laboratory, having sample preparation step being completed in a short time, reducing waste, reducing by-products, minimizing the use of organic solvents [17–24].

Supramolecular solvents (SUPRAS) are new generation, environmentally friendly solvent systems frequently used in microextraction studies. SUPRAS are nano-structured solvents and have a structure that does not mix with water. Consisting of amphiphilic aggregates, SUPRAS is formed by dispersing the reverse micelle aggregates of alkanols in a mixture such as tetrahydrofolate (THF)/water. SUPRAS increases extraction efficiency by interacting with molecules and decreasing the extraction time. The amount of mixing alkanol and THF used in the formation of SUPRAS is crucial [25–32]. To the best of our knowledge, microextraction studies of Sudan I with supramolecular solvents have not been performed before.

The aim of this study is to develop a method for enriching Sudan I with liquid phase microextraction method and determining it by UV-Vis spectrophotometer. Important parameters such as pH value, SUPRAS volume, THF volume, ultrasonic bath time and centrifugation time have been optimized. 

## 2. Materials and methods

### 2.1. Reagents and solutions

All chemicals used in the study were provided in analytical purity and were not subjected to any purification process. The ultrapure water requirement required throughout the entire study was provided by Milli-Q Millipore Direct 16 (Millipore, Bedford, MA, USA). 1-decanol and tetrahydrofuran (THF) were supplied from Sigma (St. Louis, MO, USA). 1000 mg/L solution and required concentrations of Sudan I was prepared by dissolving in ethanol. Phosphate buffer solutions pH = 2.0–4.0, acetate buffer solution with pH = 5.0, phosphate buffer solutions pH = 6.0–7.0 were prepared and used for pH value adjustments.

### 2.2. Instruments

Measurements were performed with a Hitachi UH-5300 (Tokyo, Japan) double beam spectrophotometer. pH value measurements of both model solutions and real samples were provided by the WTW ProfiLine pH 3310 portable pH Meter (Xylem Group, Weilheim, Germany). Hettich Rotofix 32A (Buckinghamshire, England) centrifuge was used to separate the SUPRAS phase and the wastewater phase. Bandelin Sonorex DT-255 (Berlin, Germany) ultrasonic water bath was used to form nano-sized SUPRAS aggregates.

### 2.3. Test procedure

**Figure 1:**
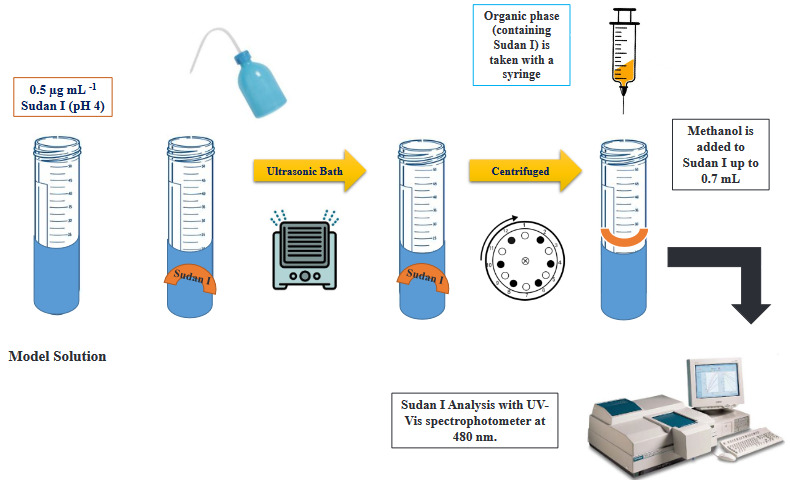
Schematic summary of the method developed.

As described in Figure 1, 100 µL of 50 mg/L Sudan I was added to a 50 mL conical bottom centrifuge tube. After 15 mL model solution was prepared by adding 2 mL of pH 4.0 phosphate buffer and distilled water. Then, 100 µL of 1-decanol and 200 µL of THF were added to the model solution to obtain the SUPRAS phase. In order to create nano or molecular micelles, the model solution exposed to ultrasonic vibration for 4 min. Then, the cloudy model solution centrifuged for 2 min. SUPRAS phase containing Sudan I remaining in the upper phase and the wastewater phase was separated with a syringe. Afterwards, the SUPRAS phase was completed to 0.7 mL with methanol, and measurements were made in a UV-Vis spectrophotometer set at 480 nm.

### 2.4. Application

Natural water samples and chili were used in the verification study of the developed method. Supramolecular solvent-based microextraction method was applied directly to natural waters. The chili sample was weighed as certain amount. Then, ethanol was added to the weighed sample and stirred for a while so that the dyestuff passed into the solvent phase. Then, sample was taken and diluted at the relevant pH value, and the developed method was applied.

## 3. Results and discussion

### 3.1. Influence of pH value

**Figure 2:**
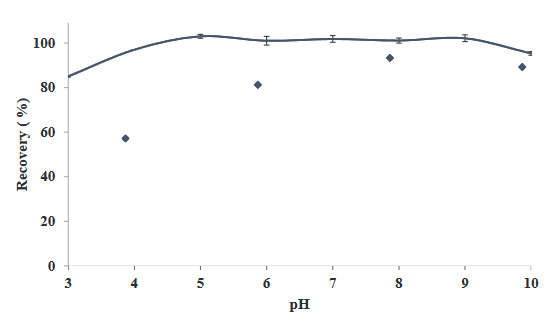
Influence of pH value of extraction recovery (N = 3).

The pH value of the medium is very important in terms of the transition of analytes to the extraction phase and, thus, extraction efficiency [33–38]. pH value of the model solution formed for this purpose was adjusted using buffer solutions varying between 3.0–10, and the remaining steps of the method were applied (Figure 2). As a result of the observations, quantitative results were observed between pH = 4.0 and 10, and the study was almost independent of pH value. In this context, pH value of 4.0 was chosen, and this value was used for the rest of the study.

### 3.2. Effects of 1-decanol and THF volume

**Figure 3:**
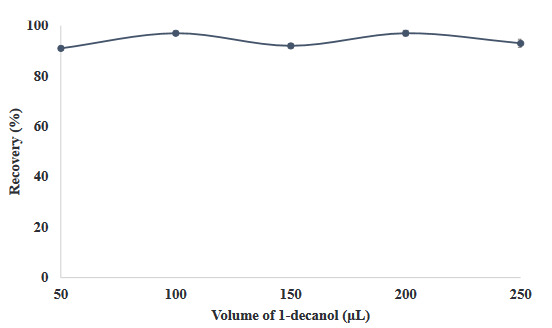
The effect of 1-decanol on the recovery yield efficiency of the developed method (N = 3).

1-decanol, a component of SUPRAS, was added to the medium in volumes ranging from 50 µL to 250 µL after the model solutions were formed. The results are shown in Figure 3, and the recovery values are quantitative when added to medium volumes ranging from 100 µL to 250 µL. In this regard, the remainder of the study was continued in the presence of 100 µL of 1-decanol.

**Figure 4:**
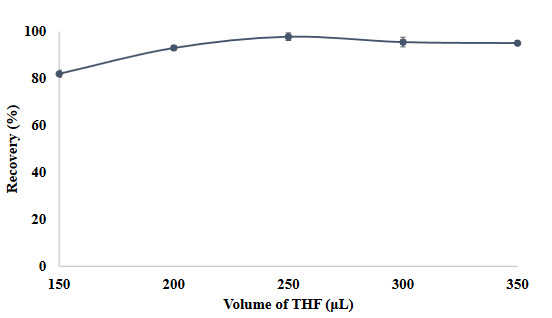
The effects of THF volume on the recovery value of the method (N = 3).

THF, another component of the SUPRAS solvent system, was added to the obtained model solution in different volumes. It is seen that quantitative values were obtained at volumes between 200 µL and 350 µL of THF added in volumes ranging from 150 µL to 350 µL (Figure 4). With the use of less reagents as a principle for this purpose, the rest of the study continued with 200 µL of THF.

### 3.3. Influence of ultrasonication and centrifugation

**Figure 5:**
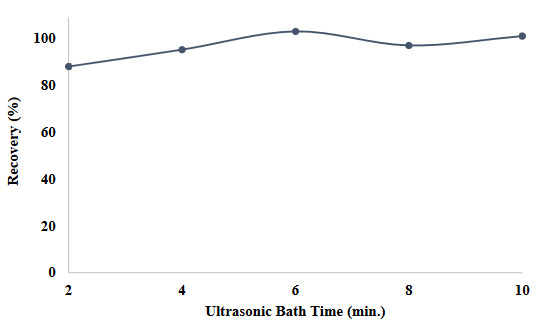
Influences of ultrasonic bath time on the recovery value of the method (N = 3).

After the model solution was formed and SUPRAS was added, the model solution was subjected to ultrasonic vibration to form reverse micelles in nano or molecular sizes. For this purpose, the effects on the recovery efficiency of the method were investigated by applying ultrasonic vibration to the model solution obtained for 2 to 10 min (Figure 5). As a result of the observations, quantitative recovery was observed in the studies between 4 and 10 min, and 4 min of ultrasonic interaction was preferred for the rest of the study.

**Figure 6:**
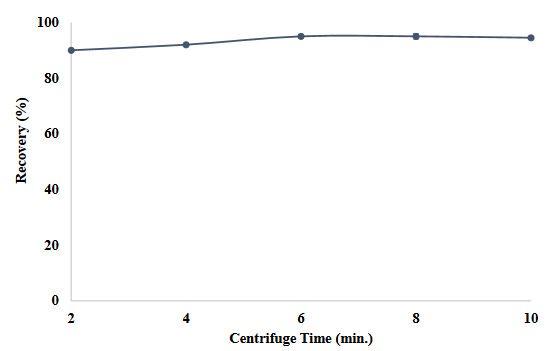
Effects of centrifuge time on the recovery value of the method (N = 3).

Centrifugation time is another factor affecting the recovery efficiency. In the absence of sufficient centrifugation, phase separations are not clear. For this reason, the obtained model was subjected to a centrifugation process after adding SUPRAS to the solution. In this context, a process between 2 and 10 min was applied in a centrifuge operating at 4000 rpm (Figure 6). As a result of the obtained observations, it was determined that the 2-min centrifugation time was sufficien and this value was used for the rest of the study.

### 3.4. Effects of sample volume

Obtaining a high preconcentration factor is directly related to the volume of the model solution. In this context, the model solution volume, which started as 10 mL, was increased up to 50 mL. As a result, quantitative results were obtained until 15 mL, and the efficiency of extraction in higher volumes decreased. For this purpose, 15 mL model solution volume was used in the rest of the study. The final volume was calculated as 0.7 mL, and the preconcentration factor was determined as 21.4.

### 3.5. Interference effects

Species that may show possible interference effects that may be found in real samples were added to the model solution medium at certain concentrations [39-43], and their effects on the recoveries of the analyte were investigated. In this context, cations, anions and dyestuffs at different concentrations were added to the environment and the effect of these species on the recovery efficiency in the environment was investigated (Table 1). As a result of the observations, no significant negative effect was detected in the presence of the added species, and the developed method showed selectivity against Sudan I.

**Table 1:** Effects of matrix species on recovery (N = 3).Interfering SpeciesConcentration (µg mL−1)Recovery %Na+200095 ± 1K+100092 ± 1Ca2+25093 ± 1Mg2+10096 ± 2Cl-100092 ± 1F-200095 ± 1Amaranth197 ± 3Ponceau 4R197 ± 1

Interfering Species	Concentration (µg mL−1)	Recovery %
Na+	2000	95 ± 1
K+	1000	92 ± 1
Ca2+	250	93 ± 1
Mg2+	100	96 ± 2
Cl-	1000	92 ± 1
F-	2000	95 ± 1
Amaranth	1	97 ± 3
Ponceau 4R	1	97 ± 1

### 3.6. Analytical figures

Ten different blank samples were prepared to determine the detection limit (LOD) and the limit of quantification (LOQ), and the developed method was applied to these blank samples under optimum conditions. Standard deviations of ten different blank samples were taken and divided by the resulting calibration curve slope. Multiplying this value by three indicates the LOD value, and multiplying it by ten indicates the LOQ value. These values were found to be 1.74 µg/L and 5.75 µg/L, respectively. Calibration graph equation was determined as y = 0.5738 x –0.0202 (x = absorbance, y = Sudan I concentration). Preconcentration factor was expressed as 21.4. Correlation coefficient (R^2^) was determined as 0.9956.

### 3.7. Applications

The aim of the liquid phase micro-extraction method is to determine the Sudan I with a relatively easy and low-cost UV-Vis spectrophotometer. This liquid phase microextraction method developed has been verified by applying recovery studies to two different water samples and one food sample (Table 2). In this context, firstly, the method was applied directly to the real samples, then the recovery efficiency values in the real sample matrix were examined by adding certain concentrations. In this way, the method is validated in different environmental matrices.

**Table 2:** Verification of the method with addition - recovery studies (N = 3).

Sample	Added, µg/mL	Found, µg/mL	Recovery, %
Tap water	0	BDL	-
	0.5	0.54 ± 0.07	108
	1	1.01 ± 0.10	101
Van Lake water	0	BDL	-
	0.5	0.51 ± 0.06	102
	1	1.04 ± 0.07	104
Sample	Added, µg/g	Found, µg/g	Recovery, %
Chili pepper	0	9.1 ± 1.10	-
	5	14.3 ± 1.80	104
	10	19.9 ± 0.30	108

## 4. Conclusion

The SUPRAS-based liquid phase microextraction study has been proposed as an effective, easy and inexpensive method for the preconcentration and determination of Sudan I by UV-Vis spectrophotometer. The most prominent feature of this method is that it can be applied in less than 15 min. The devices such as UV-Vis spectrophotometer, ultrasonic water bath and centrifuge used in this study are inexpensive instruments that can be found in almost every laboratory. This method is also very environmentally friendly thanks to the use of less solvent compared to conventional liquid-liquid extraction and solid phase extraction. This method is comparable to other methods found in the literature and stands out with less supramolecular solvent consumption and completed in a shorter time. Instead of environmentally harmful chemicals such as carbon tetrachloride, toluene, hexane and xylene used in conventional liquid phase extraction, the method was developed by using greener solvents such as 1-decanol and THF. Comparing the analysis of dyes with the liquid phase micro-extraction method performed in the literature, it was determined that only 100–200 **μL** of extraction solvent was sufficient, and the method we developed was a greener method (Table 3). These and similar features provide the potential for this method to make it a daily analysis technique that can be used in many laboratories.

**Table 3:** Comparison of the proposed method with other methods in the literature.

Technique	Analyte	LOD (μg/L)	PF	Real samples	Instrument	Ref.
Cloud point extraction	Sudan dyes	2.0–4.0	20	Chili powder	HPLC-UV	[44]
Solid phase extraction	Sudan I - IV	4.1–5.8	-	Chili products	HPLC-DAD	[45]
Liquid phase microextraction	Sudan Orange G	3.4	40	Various samples	UV-Vis Spectrophotometer	[46]
Solid phase extraction	Sudan Orange G	0.96	-	Food samples	UV-Vis Spectrophotometer	[47]
Organic drop microextraction	Sudan I - IV	0.16–0.24	62	Chili products	HPLC-PDA	[48]
Liquid phase microextraction	Sudan dyes	0.5–1.0	92–97	Food samples	HPLC-DAD	[49]
SUPRAS-based liquid phase microextraction	Sudan I	1.74	20	Chili powder and water samples	UV-Vis Spectrophotometer	This work
